# GATA6 suppresses migration and metastasis by regulating the miR-520b/CREB1 axis in gastric cancer

**DOI:** 10.1038/s41419-018-1270-x

**Published:** 2019-01-15

**Authors:** Hao Liu, Feng Du, Lina Sun, Qingfeng Wu, Jian Wu, Mingfu Tong, Xin Wang, Qi Wang, Tianyu Cao, Xiaoliang Gao, Jiayi Cao, Nan Wu, Yongzhan Nie, Daiming Fan, Yuanyuan Lu, Xiaodi Zhao

**Affiliations:** 1 State key Laboratory of Cancer Biology, National Clinical Research Center for Digestive Diseases and Xijing Hospital of Digestive Diseases, Fourth Military Medical University, Xi’an, Shaanxi China; 2 Department of Gastroenterology, Xi’an Children’s Hospital, Xi’an, Shaanxi China; 3 Department of Geriatrics, Xijing Hospital, Fourth Military Medical University, Xi’an, Shaanxi China; 4 Department of Gastroenterology, Beijing Chao-Yang Hospital, Capital Medical University, Beijing, China; 5 Faculty of Life Science, Northwest University, Xi’an, Shaanxi China

## Abstract

Transcription factors (TFs) and microRNAs (miRNAs) are tightly linked to each other in tumor development and progression, but their interactions in gastric cancer (GC) metastasis remain elusive. Here we report a novel suppressive role of GATA6 in inhibiting GC metastasis by transactivating miR-520b. We found that GATA6 expression was significantly downregulated in metastatic GC cells and tissues and that its downregulation was correlated with a poor GC prognosis. Overexpression of GATA6 suppressed GC cell migration, invasion and metastasis both in vitro and in vivo. Luciferase reporter assays and chromatin immunoprecipitation assays demonstrated that miR-520b is a direct transcriptional target of GATA6. Moreover, miR-520b expression was positively correlated with GATA6 expression in GC tissues, and ectopic expression of miR-520b inhibited the migration and invasion of GC cells. Furthermore, cAMP responsive element binding protein 1 (CREB1) was identified as a direct and functional target of miR-520b, and GATA6 could suppress GC cell migration and metastasis via miR-520b-mediated repression of CREB1. Downregulation of GATA6 and miR-520b may partly account for the overexpression of CREB1 in GC. In conclusion, our results provide novel insight into the TF-miRNA regulatory network involved in GC metastasis. Targeting the GATA6/miR-520b/CREB1 axis may be an effective approach for GC treatment.

## Introduction

Although the incidence and mortality of gastric cancer (GC) have decreased in recent years, GC still poses a tremendous threat to human health, being the fourth most common cancer and the second leading cause of cancer-related death worldwide^1^. Because GC patients in the early stage are often asymptomatic, most are diagnosed at an advanced stage with tumor metastasis, which indeed accounts for over 90% of GC-related deaths^2^. However, the underlying molecular and cellular mechanisms of GC metastasis remain largely unknown.

GATA6 belongs to a family of zinc finger-containing transcription factors (TFs) that bind to the (A/T) GATA (A/G) consensus sequence^3^. As a lineage-restricted transcription factor, GATA6 plays an important role in embryogenesis, cell differentiation, the regulation of tissue-specific genes, and carcinogenesis^4,5^. Recent studies have indicated that GATA6 also plays important roles in tumor metastasis. In pancreatic ductal adenocarcinoma (PDAC), GATA6 suppresses metastasis by inhibiting the epithelial–mesenchymal transition (EMT) both directly and indirectly^6^. In a subset of high-grade lung adenocarcinoma and metastatic cancer cells, GATA6 expression is decreased, and recovery of its function can reduce metastasis^7^. In contrast, GATA6 is reported to promote metastasis in breast cancer^8^, cholangiocarcinoma^9^ and oral squamous cell carcinoma^10^. These studies suggest that GATA6 plays context-dependent roles in tumor metastasis and that the function and potential mechanisms of GATA6 in GC metastasis remain to be elucidated.

cAMP responsive element-binding protein 1 (CREB1) is a well-known proto-oncogenic transcription factor that functions mainly by binding to the cAMP response element and regulates genes involved in oncogenesis, such as cyclins, c-FOS, EGR-1, BCL2, and MMP13^11^. Accumulating evidence suggests that CREB1 promotes tumorigenesis and is overexpressed in numerous human cancers, including breast cancer, mesothelioma, ovarian cancer, and prostate cancer^12^. In GC, CREB1 promotes the proliferation, migration and metastasis of GC cells and is overexpressed in over 90% of GC samples^13–15^. However, the mechanisms resulting in the overexpression of CREB1 in GC still require further investigation.

miRNAs are 18–24 nucleotide single-stranded RNA molecules that can inhibit the translation or promote the degradation of target mRNAs by binding to their 3’-untranslated regions (UTRs)^16^. Many studies have substantiated the critical role of miRNAs in the process of tumor metastasis, either as oncogenes or tumor suppressor genes^17^. Our previous study and other studies demonstrated that miRNAs are a class of important transcriptional targets of TFs and play a critical role in TF-mediated metastasis^18,19^. It has been reported that GATA6 could impact cell toxicity by regulating the expression of miR-30 in cardiomyocytes exposed to doxorubicin^20^. However, it remains unknown whether GATA6 could also play a role in GC metastasis by regulating certain miRNAs.

Here we found that GATA6 was downregulated in metastatic GC tissues and demonstrated that GATA6 could suppress GC cell migration, invasion, and metastasis both in vitro and in vivo. GATA6 could modulate GC metastasis through transactivation of miR-520b. CREB1 was further identified as a direct and functional target of miR-520b. Collectively, our results provide novel insight into GC metastasis involving the GATA6/miR-520b/CREB1 regulatory axis.

## Results

### GATA6 is downregulated in metastatic GC cells and tissues

**Fig. 1:**
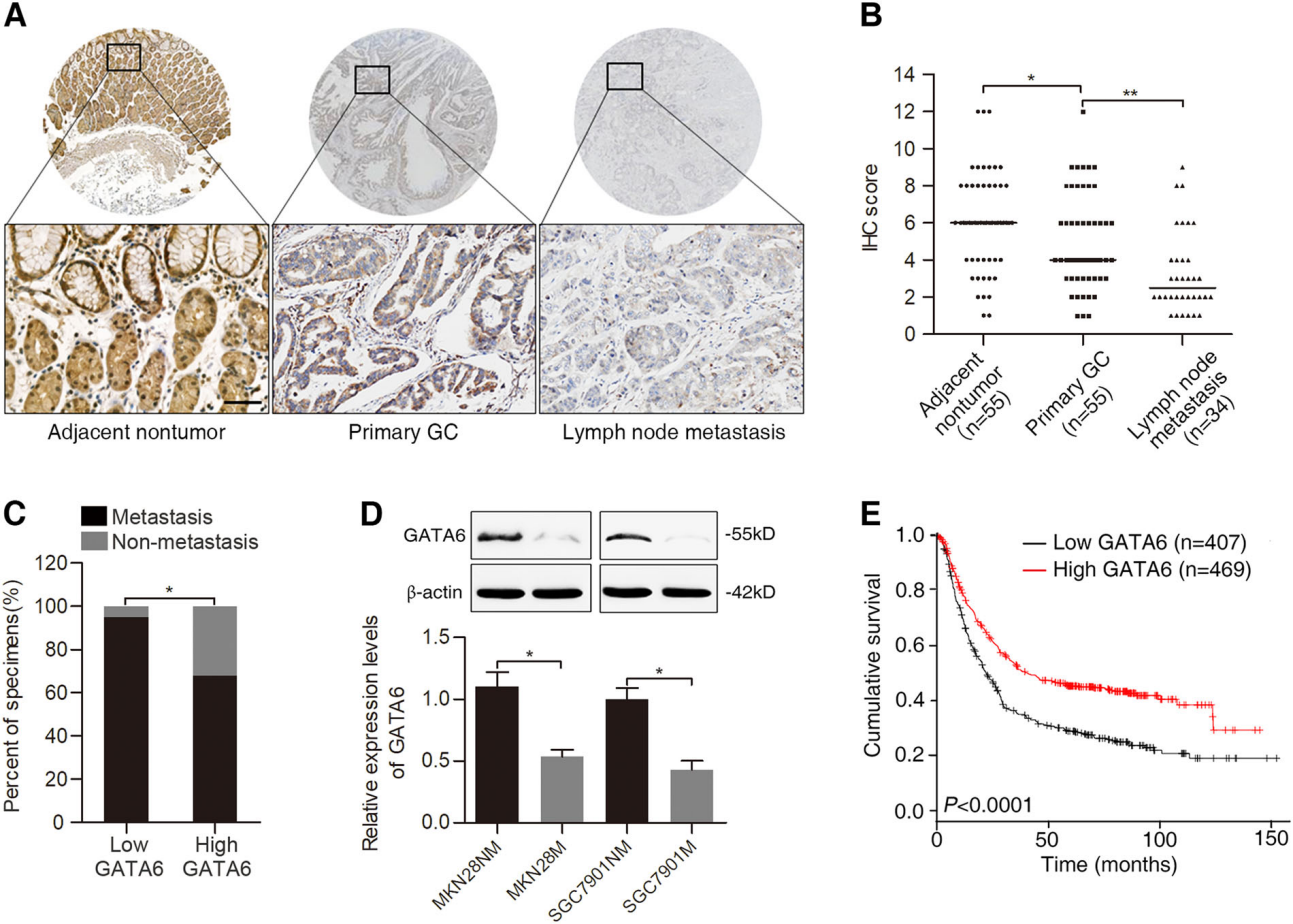
GATA6 is downregulated in metastatic GC tissues and cells and indicates a poor prognosis. **A** Representative images of GATA6 expression in adjacent nontumor tissues, primary GC tissues and lymph node metastases detected by IHC staining. Scale bars represent 100 μm. **B** IHC scores of GATA6 expression in adjacent nontumor tissues, primary GC tissues and lymph node metastases. **C** Association between GATA6 expression and occurrence of metastasis in GC specimens. **D** Western blot analysis (up) and real-time PCR analysis (down) of GATA6 expression in low- and high-metastatic GC cell lines. **E** Kaplan–Meier analysis of the correlation between GATA6 expression and the OS of patients with GC. **p < 0.01, *p < 0.05

To explore the potential role of GATA6 in GC metastasis, we first examined its expression in tissue microarrays containing samples from 34 cases of lymph node metastases, 55 cases of GC and paired adjacent nontumor tissues. Immunohistochemistry (IHC) results showed that GATA6 was primarily localized in the nucleus of glandular cells from the bottom to the top of the normal stomach epithelium and was significantly downregulated in metastatic GC tissues compared with primary GC tissues and adjacent nontumor tissues (Fig. 1A, B). The incidence of metastasis was significantly higher in the group with low GATA6 expression compared with the group with high GATA6 expression (Fig. 1C). Further, we assessed the expression of GATA6 in two pairs of low- and high-metastatic GC cell lines, MKN28NM vs MKN28M and SGC7901NM vs. SGC7901M. Compared with the low-metastatic MKN28NM and SGC7901NM cells, the high-metastatic counterparts MKN28M and SGC7901M cells exhibited relatively lower expression of GATA6 (Fig. 1D). In addition, correlation analysis showed that low-level GATA6 expression in GC tissues was significantly associated with a more aggressive tumor phenotype (Table 1). We investigated the prognostic value of GATA6 via the Kaplan–Meier plotter database, which includes 1,065 GC patients with a mean follow-up of 33 months^21^. The results showed that GC patients with low GATA6 expression exhibited significantly shorter overall survival (OS) than those with high GATA6 expression (Fig. 1E). Together, these data suggest that GATA6 is downregulated in GC metastatic tissues and cells and may play a suppressive role in GC metastasis.

**Table 1:** Correlation of GATA6 expression and patients’ clinicopathological variables in GC tissues

Variables	Expression of GATA6			*p*-value
	ALL cases (*n* = 77)	Low expression (*n* = 41)	High expression (*n* = 36)	
Age (years)				0.613
≤50	21	10	11	
≥50	56	31	25	
Gender				0.642
Male	49	25	24	
Female	28	16	12	
Tumor size (cm)				0.174
≤5	34	15	19	
>5	43	26	17	
Grade of differentiation				0.037
G1	6	4	2	
G2	33	12	21	
G3	38	25	13	
Tumor invasion				0.014
T1	14	5	9	
T2	9	2	7	
T3	44	29	15	
T4a/b	3	3	0	
Lymph node status				0.008
N0	12	2	10	
N1	8	3	5	
N2	18	13	5	
N3a/b	32	21	11	
Distant metastasis				0.012
M0	57	25	32	
M1	13	11	2	

### GATA6 inhibits GC cell migration and metastasis in vitro and in vivo

**Fig. 2:**
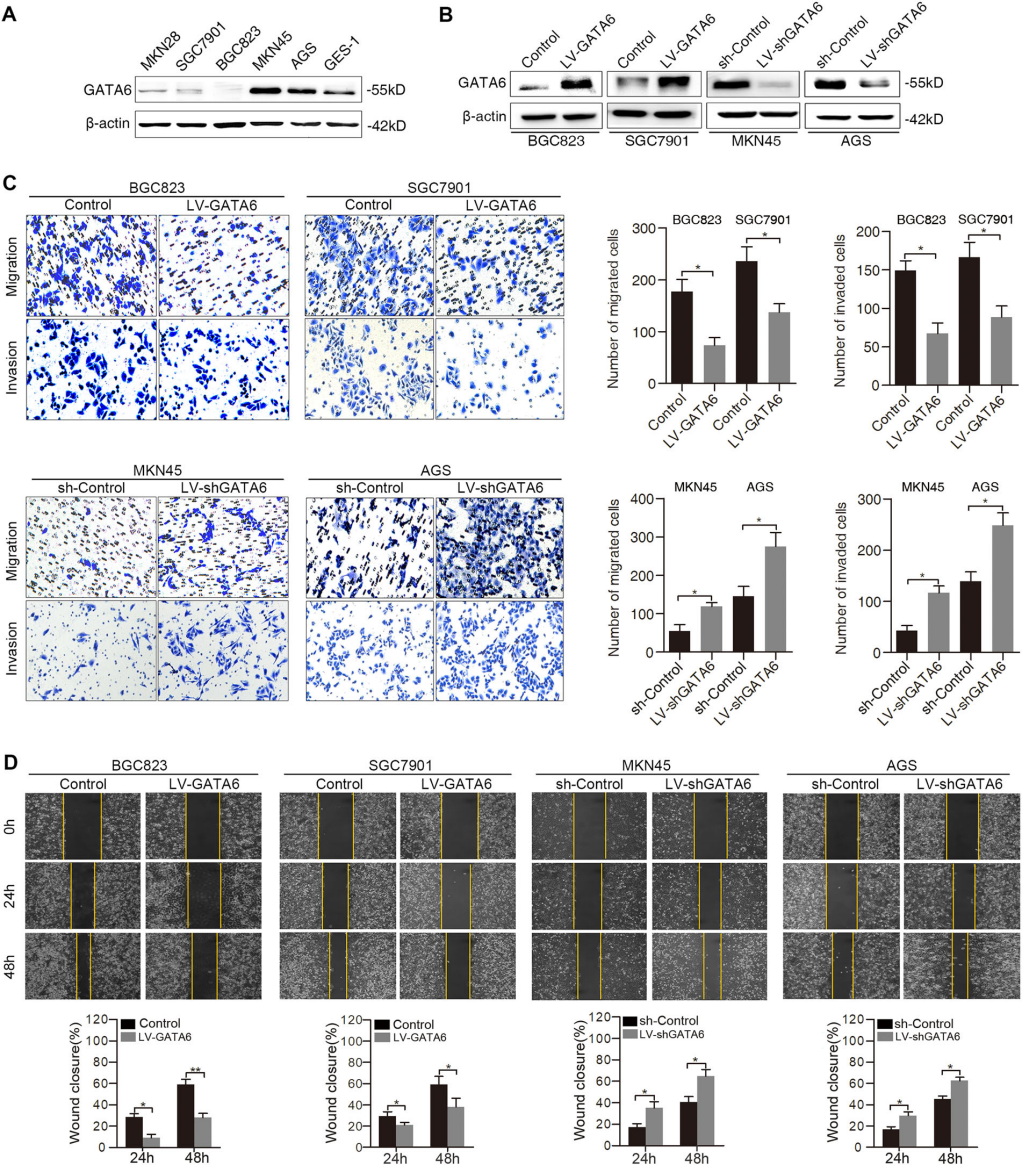
GATA6 suppresses GC cell migration and invasion in vitro. **A** Western blot analysis of GATA6 expression in GC cell lines and an immortalized gastric epithelial cell line, GES-1. **B** Western blot analysis of GATA6 expression in GC cells after infection with LV-GATA6, LV-shGATA6, and empty vector controls. **C** Transwell assays showing the migration and invasion abilities of the indicated GC cells. **D** Wound-healing assays showing the migration abilities of the indicated GC cells. The percentage of wound closure was normalized to 0 h. **p < 0.01, *p < 0.05

To determine the role of GATA6 in GC metastasis, GATA6 expression was first examined in several GC cell lines as well as GES-1, an immortalized gastric epithelial cell line (Fig. 2A). We found that GATA6 expression was downregulated in MKN28, SGC7901 and BGC823 cells compared with GES-1 cells. Then, lentiviral GATA6 (LV-GATA6) was stably transduced into BGC823 and SGC7901 cells, and GATA6 shRNA (LV-shGATA6) was stably transduced into MKN45 and AGS cells, with empty vector transduction as a control. Overexpression and silencing of GATA6 were confirmed by Western blotting (Fig. 2B). As shown by transwell assays, upregulation of GATA6 significantly decreased the migration and invasion abilities of BGC823 and SGC7901 cells, while downregulation of GATA6 increased the migration and invasion abilities of MKN45 and AGS cells (Fig. 2C). Consistently, wound-healing assays demonstrated that ectopic expression of GATA6 inhibited cell migration, while silencing of GATA6 enhanced cell migration (Fig. 2D).

**Fig. 3:**
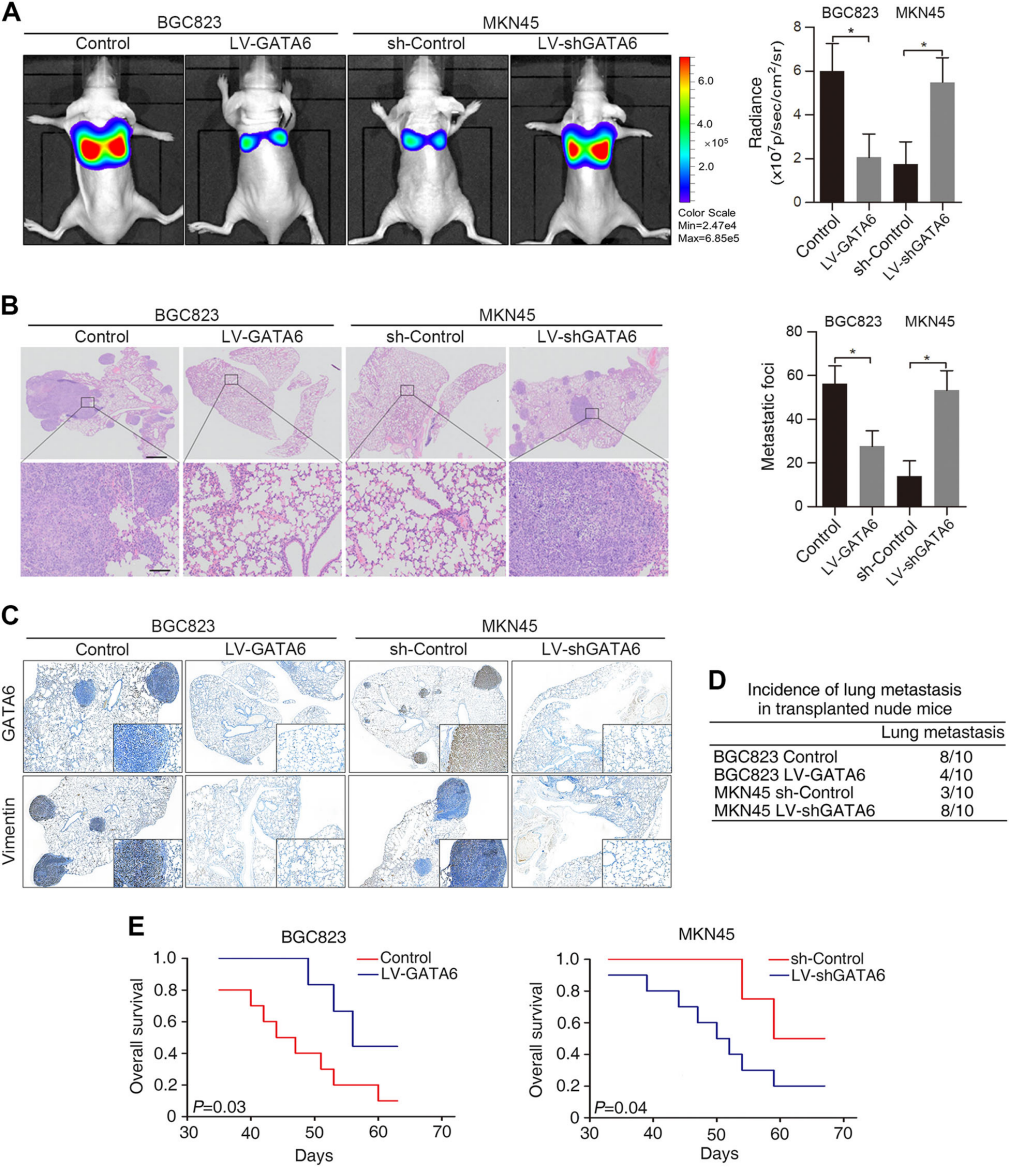
GATA6 suppresses GC cell metastasis in vivo. **A** Representative bioluminescence images of the different groups are shown at 8 weeks after tail vein injection (left). The radiance was collected and calculated (right). **B** Representative hematoxylin and eosin (H&E) staining images of lung tissue sections from different groups (left). The number of lung metastatic foci was calculated (right). Scale bars: 200 μm (top) and 50 μm (bottom). **C** Representative human GATA6 and Vimentin IHC staining of whole lung sections. Bars: (main) 200 µm; (insets) 50 µm. **D** Incidences of lung metastases of each group. **E** OS time of the nude mice in each group. *p < 0.05

To further validate our findings in vivo, we injected BGC823 LV-GATA6 and MKN45 LV-shGATA6 cells through the tail vein of nude mice. Overexpression of GATA6 resulted in reduced bioluminescence intensity in the lung (Fig. 3A), fewer metastatic lung nodules (Fig. 3B) and a lower incidence of lung metastasis in the BGC823 LV-GATA6 group (Fig. 3D). The resulting metastatic lesions showed positive staining for human Vimentin by IHC (Fig. 3C). In addition, overexpression of GATA6 led to a prolonged survival time in the BGC823 LV-GATA6 group (Fig. 3E). Conversely, downregulation of GATA6 resulted in an opposite effect in the MKN45 LV-shGATA6 group (Fig. 3A–E). Taken together, these in vitro and in vivo experiments demonstrated that GATA6 inhibits GC cell migration, invasion and metastasis.

### GATA6 transcriptionally regulates miR-520b expression by directly targeting the miR-520b promoter

**Fig. 4:**
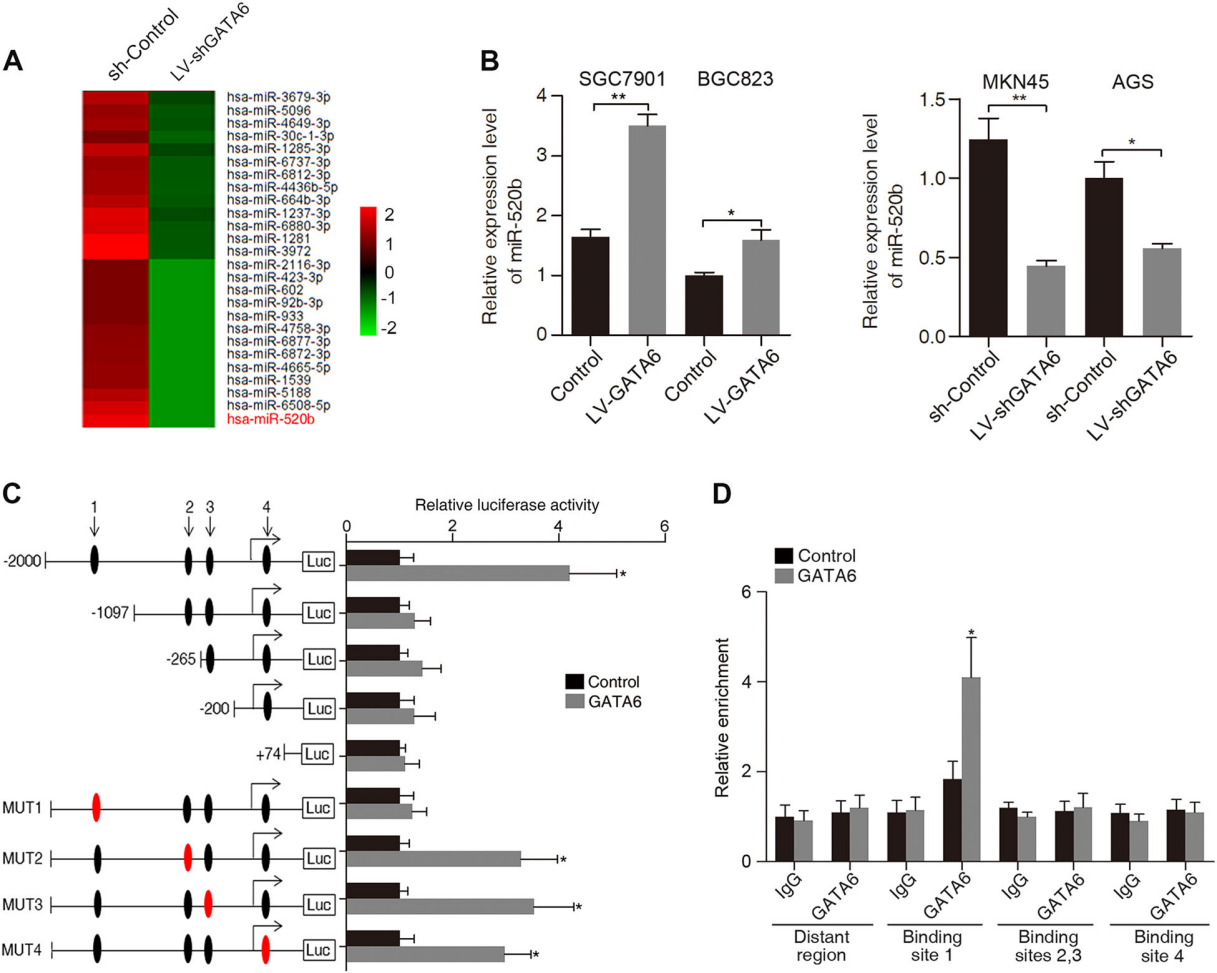
GATA6 transactivates miR-520b in GC cells. **A** Heatmap showing miRNAs downregulated upon GATA6 silencing in MKN45 cells. The scale bar shows color-coded differences in expression from the mean. **B** Expression of miR-520b after overexpression and downregulation of GATA6 in GC cells. **C** Serially truncated and mutated miR-520b luciferase reporter constructs were transfected into SGC7901 LV-GATA6 (GATA6) and SGC7901 LV-Control (Control) cells. Luciferase activity values were measured and analyzed. Luciferase values are normalized to the empty vector control. **D** ChIP assays demonstrated the direct binding of GATA6 to binding site 1 of the miR-520b promoter in SGC7901 cells. Data are presented as the relative enrichment normalized to control IgG. **p < 0.01, *p < 0.05

To investigate whether GATA6 can modulate GC metastasis by regulating certain miRNAs, we compared differentially expressed miRNAs when GATA6 was silenced in MKN45 cells using a miRNA microarray. In total, 91 miRNAs were significantly changed after GATA6 downregulation in MKN45 cells, with the largest decrease for miR-520b (Fig. 4A and Supplementary Fig. S1A). Real-time PCR confirmed that upregulation of GATA6 increased miR-520b expression, while downregulation of GATA6 reduced its expression, suggesting a promoting effect of GATA6 on miR-520b (Fig. 4B). Four putative GATA binding sites, (G/A) GATA (A/T), were identified in the miR-520b promoter region (Fig. 4C). Site-directed mutagenesis and serial deletion analysis of the miR-520b promoter identified that GATA6-binding site 1 (−1103bp upstream of the transcription start site (TSS)) was critical for GATA6-mediated transcriptional activation (Fig. 4C). Further chromatin immunoprecipitation (ChIP) assays showed that GATA6 proteins bound directly to GATA6-binding site 1 in GC cells (Fig. 4D). These results indicate that miR-520b is a direct target of GATA6.

### Ectopic expression of miR-520b regulates GC cell migration and invasion

**Fig. 5:**
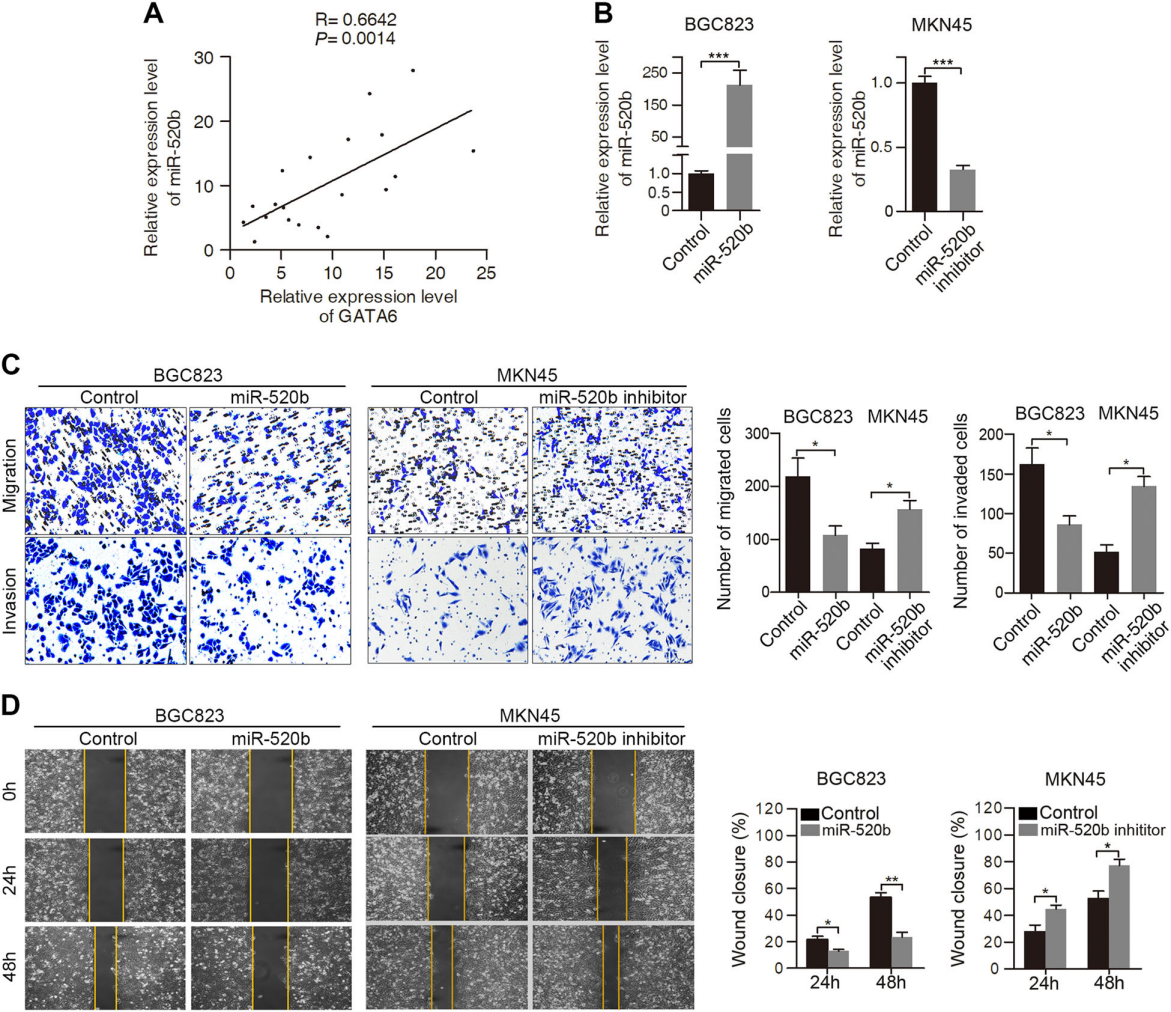
miR-520b inhibits the migration and invasion of GC cells. **A** Positive correlation between GATA6 and miR-520b expression in GC tissues (*n* = 20). **B** Transfection of miR-520b mimics and inhibitors into BGC823 cells and MKN45 cells, respectively. **C** Transwell assays of BGC823 and MKN45 cells transfected with miR-520b mimics or inhibitors and negative controls. **D** Wound-healing assays of BGC823 and MKN45 cells transfected with miR-520b mimics or inhibitors and negative controls. The percentage of wound closure was normalized to 0 h. ***p < 0.001, **p < 0.01, *p < 0.05

We next investigated whether there is a correlation between GATA6 expression and miR-520b expression in GC tissues. Statistical analysis revealed that miR-520b expression levels positively correlated with GATA6 expression (Fig. 5A). To evaluate the role of miR-520b in GC cell migration and invasion, we transfected miR-520b mimics and inhibitors into BGC823 cells and MKN45 cells (Fig. 5B). The transwell assays revealed that overexpression of miR-520b suppressed migration and invasion, while inhibition of miR-520b significantly promoted the migration and invasion of GC cells (Fig. 5C). Similarly, the wound-healing assays showed that miR-520b upregulation led to a decrease in cell migration, whereas miR-520b inhibition exhibited the opposite effect (Fig. 5D). Collectively, these results indicate that miR-520b inhibited the migration and invasion of GC cells.

### miR-520b suppresses GC cell migration and invasion by directly targeting CREB1

**Fig. 6:**
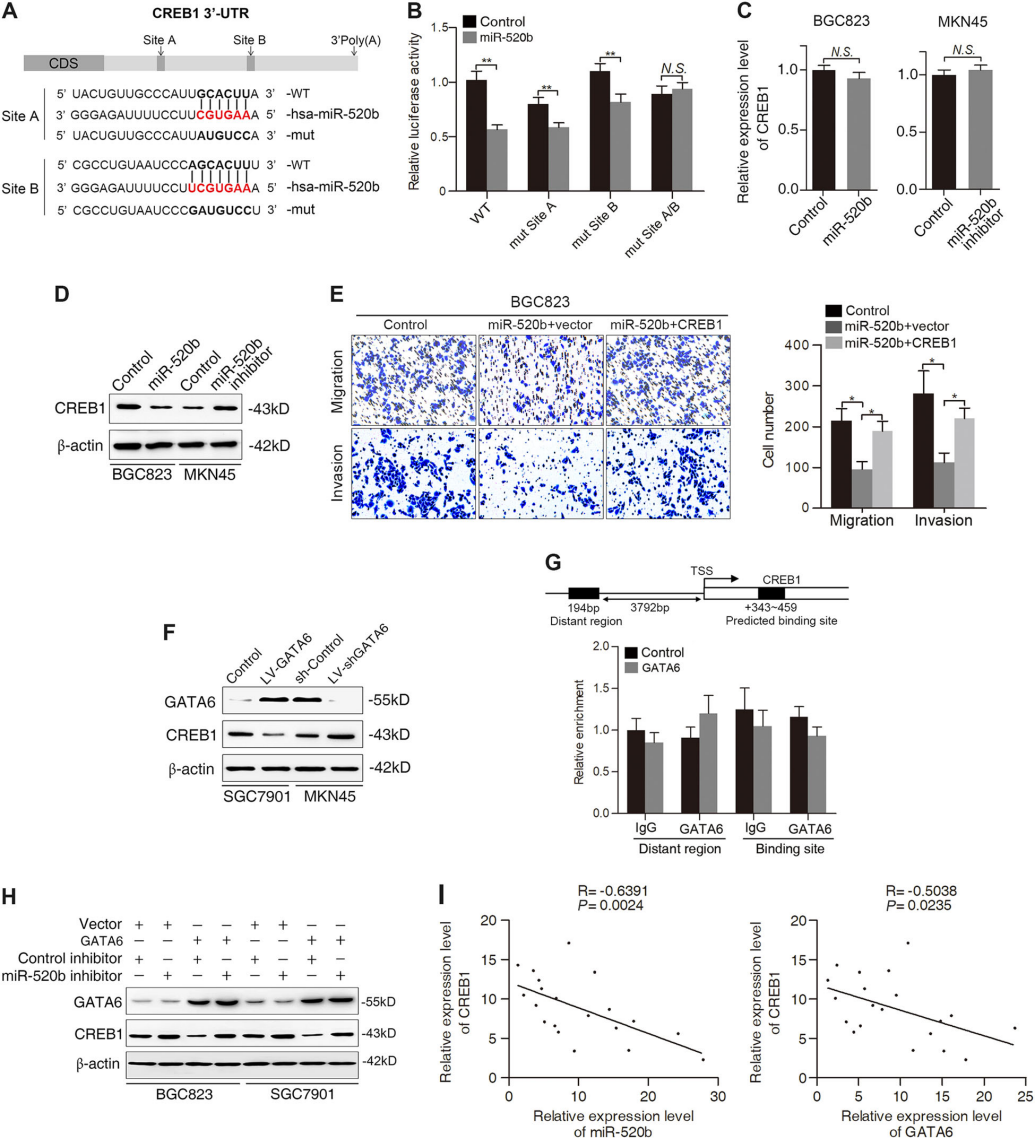
CREB1 is a direct and functional target of miR-520b. **A** Diagram of the CREB1 3′-UTR luciferase reporter constructs containing wild-type or mutant miR-520b binding sites. **B** Relative luciferase activity in BGC823 cells cotransfected with wild-type or mutated reporter plasmids and miR-520b or controls. **C** PCR analysis of *CREB1* mRNA expression in BGC823 and MKN45 cells transfected with miR-520b mimics and inhibitors, respectively. **D** Western blot analysis of CREB1 protein levels in the indicated GC cells. **E** Transwell assays of the migration and invasion abilities of BGC823 cells transfected with miR-520b, the CREB1 plasmid (CREB1) or the control plasmid vector (vector). **F** Western blot analysis of GATA6 and CREB1 expression in SGC7901 and MKN45 cells transduced with LV-GATA6 and LV-shGATA6 and an empty control vector, respectively. **G** Schematic structure of the CREB1 upstream promoter containing one GATA6-binding site. ChIP assays showing that GATA6 could not bind upstream of the CREB1 promoter region in SGC7901 cells. **H** Western blot analysis of GATA6 and CREB1 expression in BGC823 and SGC7901 cells infected with GATA6 or vector control and transfected with miR-520b inhibitor or control. **I** Negative correlation between miR-520b or GATA6 and CREB1 in GC tissues (*n* = 20). **p < 0.01, *p < 0.05. N.S. not significant (*p* > 0.05)

To figure out the underlying mechanism by which miR-520b inhibits GC cell migration and invasion, we employed several computational methods to identify potential targets. Among these targets, CREB1 was of particular interest because it was reported to be upregulated in GC and to be involved in the promotion of GC metastasis^22^. To determine whether CREB1 is a direct target of miR-520b, we performed luciferase reporter assays in BGC823 cells. The wild-type or mutant 3′-UTR of the CREB1 mRNA was inserted into a luciferase reporter vector (Fig. 6A). As revealed by the luciferase reporter assays, miR-520b overexpression suppressed the wild-type CREB1 3′-UTR reporter but did not affect the mutant CREB1 3′-UTR luciferase reporter (Fig. 6B). Real-time PCR assays showed that the mRNA levels of CREB1 remained unaltered when miR-520b was overexpressed or inhibited (Fig. 6C). However, Western blot analysis demonstrated that overexpression of miR-520b greatly decreased CREB1 protein levels in BGC823 cells, whereas downregulation of miR-520b increased CREB1 expression in MKN45 cells (Fig. 6D), which indicates that miR-520b regulates CREB1 at the post-transcriptional level.

To determine whether downregulation of CREB1 is responsible for the decrease in cell migration and invasion upon miR-520b overexpression, we transfected BGC823 cells with miR-520b and then with a CREB1 construct or control vector. The transwell assays revealed that CREB1 expression significantly rescued the miR-520b-induced decrease in GC cell migration and invasion (Fig. 6E). Taken together, these results indicate that CREB1 is both a direct and functional target of miR-520b.

### GATA6 regulates CREB1 expression through a miR-520b-dependent manner

Since GATA6 could transactivate miR-520b and since miR-520b could post-transcriptionally repress CREB1, we thus asked whether GATA6 affects CREB1 level in a miR-520b-dependent manner. Western blot analysis showed that overexpression of GATA6 downregulated the expression of CREB1, whereas silencing of GATA6 enhanced the expression of CREB1 (Fig. 6F). To clarify the mechanisms underlying the GATA6-induced downregulation of CREB1, we first investigated whether GATA6, as a transcription factor, could transcriptionally repress CREB1 expression. Bioinformatics analysis identified that there was one GATA6-binding site in CREB1 promoter region. However, subsequent ChIP assays demonstrated that GATA6 could not bind to the CREB1 promoter directly, indicating that GATA6 did not regulate CREB1 expression at transcriptional level (Fig. 6G). We therefore verified whether GATA6 indirectly regulates CREB1 expression through miR-520b. To this end, we cotransfected GATA6 vector, miR-520b inhibitors and negative control into BGC823 and SGC7901 cells. Transfection with GATA6 distinctly inhibited CREB1 expression, whereas miR-520b inhibition abrogated the CREB1 repression induced by ectopic GATA6 expression (Fig. 6H), suggesting that GATA6 might suppress CREB1 via miR-520b. In addition, negative correlations were observed between CREB1 and miR-520b or GATA6 expression in GC tissues (Fig. 6I). Collectively, these results suggest that GATA6 may regulate the expression of CREB1 through miR-520b.

## Discussion

Dysregulation of certain TFs plays a significant role in the process of tumor metastasis in various cancer types^23^. In the present study, we found for the first time that GATA6 was significantly downregulated in metastatic GC cells and tissues and that GATA6 was correlated with poor prognosis of GC patients. In vitro and in vivo experiments demonstrated that overexpression of GATA6 suppressed the migration, invasion and metastasis of GC cells. Regarding the mechanism, we identified that GATA6 inhibited GC cell metastasis through the GATA6/miR-520b/CREB1 pathway.

The role of GATA6 in cancer has gained increasing attention recently, but conflicting evidence exists, even for the same tumor type. For instance, in CRC, GATA6 serves as an oncogene by repressing BMP expression and enhancing Lgr5 expression^24,25^. In contrast, a tumor suppressive role of GATA6 in cetuximab resistance was demonstrated by decreasing the expression of MIR100HG^26^. Similarly, in PDAC, GATA6 was amplified in a subset of pancreatic tumors, and overexpression of GATA6 increased pancreatic cancer proliferation^27,28^. However, the classic PDAC subtype, which showed a better outcome, had a relatively high expression of GATA6, and GATA6 was proven to be a suppressor in the process of PDAC metastasis^6,29^. These discrepancies further support the idea of a complex role of GATA6 in tumor progression and strongly suggest that the role of GATA6 in cancer is stage- and context-dependent. A previous study reported that GATA6 promoted proliferation and was upregulated in GC^30^. However, our data indicated that GATA6 expression was significantly reduced in metastatic GC cells and tissues. In addition, ectopic expression of GATA6 dramatically suppressed GC cell metastasis in vitro and in vivo. The differences in the cell lines used and the stage studied may be responsible for the contradiction, which highly supports the notion that lineage-survival TFs such as GATA6 exert context-dependent effects in cancer^31^.

TFs and miRNAs, which are both critical gene regulators, are tightly linked to each other^32^. The biogenesis and expression of miRNAs are transcriptionally regulated by a series of TFs, while the function and expression of TFs are post-transcriptionally regulated by miRNAs. The important role of TF-miRNA connections in the gene regulatory network has been reported in many aspects of cancer progression, such as carcinogenesis^33,34^, metastasis^35^ and drug resistance^36^. In this study, we found that GATA6 inhibits GC cell metastasis by directly regulating miR-520b. However, miR-520b failed to affect the expression of GATA6 (data not shown), thus excluding the possibility of a regulatory feedback loop. Previous studies demonstrated that miR-520b acts as a tumor suppressor in glioma^37^, hepatoma^38^ and lung cancer^39^. Our study verified that miR-520b plays a pivotal role in GATA6-mediated suppression of the GC metastatic process, which is in line with a previous report^40^. It should be noted that GATA6 may suppress GC metastasis via transcriptional targets other than miR-520b. Indeed, among the miRNAs that are differentially expressed upon GATA6 silencing, we also identified significant changes in other metastasis-related miRNAs, such as miR-92b, miR-181, miR-200, and miR-151. GATA6 likely inhibits GC metastasis by transactivating or repressing these targets, which will be investigated in our future study.

For the downstream target that mediated the GATA6/miR-520b-regulated inhibition of GC metastasis, we identified CREB1 as a direct target of miR-520b. Mounting evidence indicates that CREB1 exhibits oncogenic functions in cancer progression. For instance, CREB1 was demonstrated to promote the development of acute myeloid leukemia^41^ and lung cancer^42^ and was responsible for the aberrantly high levels of oncogenic TGFβ2 in glioblastoma^43^. In GC, CREB1 was overexpressed and correlated with lymph node metastasis, distant metastasis and poor prognosis^22^. However, the mechanisms leading to CREB1 overexpression in GC are poorly understood. Our present study established that CREB1 was a direct and functional target of miR-520b and that GATA6 was capable of regulating the expression of CREB1 through miR-520b. In addition, both miR-520b and GATA6 showed a negative correlation with CREB1 in GC tissues, indicating that the aberrant expression of CREB1 in GC may be partly due to the dysregulation of GATA6 and miR-520b. To our knowledge, this is the first evidence in GC that one TF (GATA6) can regulate the expression of another TF (CREB1) indirectly through a miRNA, which may contribute to a better understanding of the TF regulatory network in cancer metastasis.

In summary, our study revealed the previously unreported role of GATA6 in suppressing GC metastasis and its potential mechanisms. Overexpression of GATA6 inhibits GC metastasis by transactivating miR-520b, which results in downregulation of CREB1. The GATA6/miR-520b/CREB1 axis represents a novel component of the mechanisms underlying GC metastasis and offers a candidate therapeutic target for GC treatment.

## Materials and methods

### Cell lines

The human SGC7901, BGC823, AGS, MKN45 and MKN28 GC cell lines and the immortalized gastric epithelial cell line GES-1 were purchased from the Cell Resource Center of the Chinese Academy of Sciences (Shanghai, China). The high-metastatic cell lines MKN28M and SGC7901M and the low-metastatic cell lines MKN28NM and SGC7901NM were established as previously described^44^. All cells were cultured in Dulbecco′s modified Eagle’s medium (DMEM, Thermo Fisher Scientific Gibco, Beijing, China) supplemented with 10% fetal bovine serum (Gibco BRL) and incubated in 5% CO_2_ at 37 °C.

### Tissue specimens

GC tissue microarray chip containing 25 adjacent nontumor tissue, 25 paired primary GC tissue and 16 GC lymph node metastatic tissue samples was purchased from Outdo Biotech (Shanghai, China). GC tissue microarray chip containing 30 GC tissues paired with matched adjacent nontumor tissues, 22 gastric malignant tissues and 18 GC lymph node metastatic tissue samples was obtained from Alenabio (Xi’an, China). Twenty samples of primary GC tissues were obtained from patients who had undergone gastric cancer surgery at Xijing Hospital, Xi’an, China. All patients provided informed consent. This study was approved by the Hospital’s Protection of Human Subjects Committee.

### RNA extraction and real-time PCR

Total RNA was extracted using the RNeasy Plus Mini Kit (50) (Qiagen, Hilden, Germany), and miRNA was extracted using the miRNeasy Mini Kit (Qiagen) according to the manufacturer’s instructions. Then, cDNA was synthesized using a PrimeScript RT reagent kit (TaKaRa, Dalian, China). The SYBR Premix Ex Taq II (TaKaRa) was used to amplify the double-stranded cDNA of interest. qPCR primers for miR-520b and U6 were purchased from RuiBo Bio (Guangzhou, China). qPCR primers for GATA6, CREB1 and ACTB (β-actin) were synthesized by TaKaRa (Dalian, China). The levels of U6 and ACTB were used as internal controls for miRNA and mRNA, respectively. The 2^–ΔΔCt^ method was used to determine the relative expression level of RNA between groups. The primer sequences are listed in Supplementary Table S1.

### Protein extraction and Western blot analysis

The proteins were harvested with RIPA buffer (Beyotime, Shanghai, China) containing a complete protease inhibitor cocktail (Roche, Manheim, Germany). Approximately 20–50 µg of denatured protein was fractionated by SDS-PAGE and transferred to nitrocellulose membranes. The following antibodies were used: anti-GATA6 (CST #5851), anti-CREB1 (Abcam #32515) and anti-β-actin (Sigma-Aldrich A1978). Proteins were visualized using a Dura SuperSignal Substrate (Pierce, USA). Blots were scanned using a Molecular Imager ChemiDox XRS + Imaging System with Image Lab software (Bio-Rad Laboratories).

### Migration and invasion assays

For the migration assays, transfected or infected cells were suspended in serum-free DMEM, and 1 × 10^5^ cells were plated in the top chamber lined with an uncoated 8.0 µm pore membrane (EMD Millipore, Billerica, MA, USA). For the invasion assays, 1 × 10^5^ cells were plated in the top chamber coated with Matrigel (Corning, #354480, Bedford, MA, USA). Then, the chambers were inserted into a 24-well plate containing DMEM with 20% fetal bovine serum. After incubation in 5% CO_2_ at 37 °C for 24 h, cells that had migrated or invaded through the membrane were stained with 0.1% crystal violet and counted under a microscope (Olympus, Tokyo, Japan) to determine their relative numbers.

### Wound-healing assays

The Culture-Insert 2 Well (ibidi, #81176, Martinsried, Germany) was used for the wound-healing assays according to the manufacturer’s protocol. Briefly, transfected or infected cells were suspended at a concentration of 3–7 × 10^5^ cells/ml, and 70 µl of cell suspension was applied to each well. The cells were starved with serum deprivation media (with 0.1% FBS) for approximately 24 h. After appropriate cell attachment, the wounded monolayer cell was cultured with fresh serum-free DMEM, then the well was gently removed, and a scratch was made. The wounded monolayer was washed with phosphate-buffered saline (PBS) and imaged 0, 24, and 48 h after scratching using an Olympus camera system. The assays were conducted in triplicate.

### In vivo metastasis assays

Briefly, 2 × 10^6^ luciferase-tagged GC cells infected with LV-GATA6, LV-shGATA6, or the corresponding negative control were suspended in 200 µl of PBS and injected into the tail vein of nude mice supplied by the Experimental Animal Center of the Fourth Military Medical University. Five weeks after injection, D-luciferin (Xenogen, Hopkinton, MA) at 100 mg/kg was injected intraperitoneally into the mice, and bioluminescence was detected using an IVIS 100 Imaging System (Xenogen) at weekly intervals. The survival of the mice was recorded daily. Mice were sacrificed and examined for lung metastasis using standard histological examination 8 weeks after injection. The protocol for the animal studies was approved by the Fourth Military Medical University Animal Care Committee.

### Plasmid construction

The miR-520b promoter construct was generated as previously described^45^. Briefly, (−2000/ + 500) MIR520b was generated from human genomic DNA. This construct, corresponding to the sequence from −2000 to + 500 (relative to the transcriptional start site) of the 5′-flanking region of the human gene, was generated with the forward and reverse primers incorporating MluI and XhoI sites at the 5′ and 3′ ends, respectively. The MluI and XhoI sites of the pGL3-Basic Vector (Promega) were inserted for the ultimate PCR product. Constructs including a deletion of the 5′-flanking region of the miR-520b promoter ((−1097/ + 500) MIR520b, (−265/ + 500) MIR520b, (−200/ + 500) MIR520b and ( + 74/ + 500) MIR520b) were generated in manner analogous to that for the (−2000/ + 500) MIR520b construct. The QuikChange II Site-Directed Mutagenesis Kit (Stratagene, La Jolla, CA) was used to generate the constructs for site-directed mutation. All of the above constructs were verified by sequencing. All primers used are listed in Supplementary Table S1.

### Luciferase reporter assays

The miRNA 3′-UTR luciferase reporter vectors were constructed as previously described^18^. For 3′-UTR luciferase reporter assays, the indicated cells were cotransfected with miR-520b mimic or negative control (RiboBio) and the indicated wild-type or mutant psiCHECK-2-3′UTR plasmids using Lipofectamine 2000 (Thermo Fisher Scientific). For luciferase reporter assays of promoter activity, the indicated cells were cotransfected with the pGL3-MIR-520B promoter fragment, pRL-SV40 Renilla luciferase reporter, and LV-GATA6 or control. The Dual-Luciferase Assay (Promega) was used to detect Renilla and firefly luciferase activities. Renilla luciferase activity was normalized to the firefly activity and presented as the relative luciferase activity. All assays were performed in triplicate three times.

### Chromatin immunoprecipitation (ChIP)

ChIP assays were performed as previously described^26^. Briefly, the recovered supernatants were incubated with rabbit anti-GATA6 antibody (CST #5851) or an isotype control IgG (BD) for 2 h in the presence of herring sperm DNA and protein A/G magnetic beads. The DNA was recovered and subjected to PCR to amplify the GATA6-binding sites. The primers are shown in Supplementary Table S1.

### Immunohistochemistry (IHC)

IHC staining was conducted as previously described^34^. Tissue sections were deparaffinized, subjected to antigen retrieval and endogenous peroxidase inactivation and incubated with primary antibodies against GATA6 (Abcam #22600) and Vimentin (CST #5741). Then, the sections were incubated with a peroxidase-conjugated secondary antibody (Santa Cruz), followed by visualization with diaminobenzidine and image collection by a light microscope (Olympus, Japan). The final immunoactivity scores of each section were determined by two independent observers in a blinded manner according to standard procedures described previously^34^. Samples with IHC scores å 4 were determined to have high expression, and samples with IHC scores ⩽ 4 were determined to have low expression.

### Oligonucleotide transfection

The miR-520b mimic, miRNA mimic negative control, miR-520b inhibitor, and miRNA inhibitor negative control oligonucleotides were chemically synthesized and purified by RiboBio (RiboBio). The sense strand sequences of GATA6 siRNAs designed to target human cells were as follows: GATA6 siRNA no. 1, 5′-GUGGACUCUACAUGAAACUTT-3′; GATA6 siRNA no. 2, 5′-GCUCUGGUAAUAGCAAUAATT-3′; and GATA6 siRNA no. 3, 5′-GCUCAAGUAUUCGGGUCAATT-3′′. Successful knockdown of GATA6 was confirmed by Western blotting (Supplementary Fig. S1B). The siRNA no. 3 hairpin sequence was used to construct a lentiviral vector (LV-shGATA6). Transfection of the siRNA, miRNA mimic, and miRNA inhibitor was performed using RNAiMAX Reagent (Thermo Fisher Scientific) according to the manufacturer’s instructions.

### miRNA expression profiling

RNA from MKN45 sh-Control and MKN45 LV-shGATA6 cells was extracted using the miRNeasy Mini Kit (Qiagen). The Affymetrix Chip Human Array (Affymetrix, Santa Clara, CA, USA) was used to assess the miRNA expression profiles of the indicated cells as previously described^46^.

### Kaplan–Meier plotter analysis

The prognostic value of the GATA6 gene in GC was analyzed using Kaplan–Meier plotter (http://kmplot.com/analysis/)^21^. The Kaplan–Meier plotter is an online database which is capable of assessing the effect of genes on survival using cancer samples. This database includes 1,065 gastric cancer patients with a mean follow-up of 33 months. Patients with higher and lower expression of GATA6 (Probe ID: 210002_at) were segregated and analyzed using the log-rank test. All data sets were included, except GSE62254. The hazard ratio with 95% confidence intervals and log-rank p values were noted.

### Statistical analyses

All analyses were performed using SPSS software (version 22.0). The data are presented as the means ± the standard errors of the mean. Student’s *t* test (two-tailed), ANOVA (Dunnett’s or LSD post hoc test), Pearson correlation coefficients or *χ*^2^-tests were used to analyze the data according to the type of experiment. *P* < 0.05 was considered significant.

## Supplementary information

supplementary figure S1

supplementary table 1
