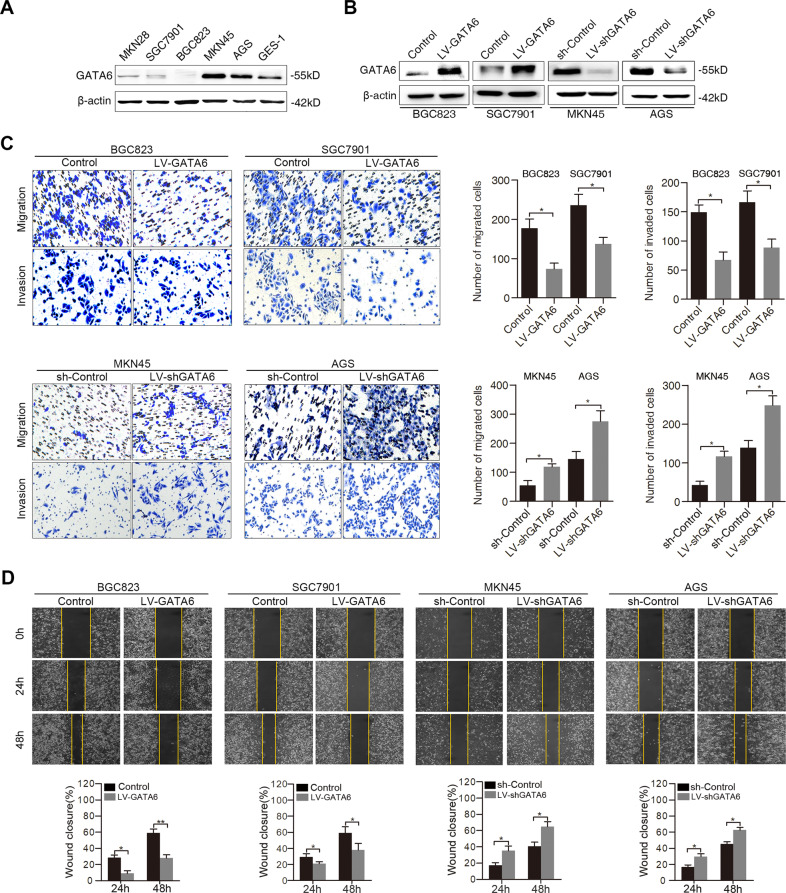# Correction to: GATA6 suppresses migration and metastasis by regulating the miR-520b/CREB1 axis in gastric cancer

**DOI:** 10.1038/s41419-022-04702-2

**Published:** 2022-03-16

**Authors:** Hao Liu, Feng Du, Lina Sun, Qingfeng Wu, Jian Wu, Mingfu Tong, Xin Wang, Qi Wang, Tianyu Cao, Xiaoliang Gao, Jiayi Cao, Nan Wu, Yongzhan Nie, Daiming Fan, Yuanyuan Lu, Xiaodi Zhao

**Affiliations:** 1 State key Laboratory of Cancer Biology, National Clinical Research Center for Digestive Diseases and Xijing Hospital of Digestive Diseases, Fourth Military Medical University, Xi’an, Shaanxi China; 2 Department of Gastroenterology, Xi’an Children’s Hospital, Xi’an, Shaanxi China; 3 Department of Geriatrics, Xijing Hospital, Fourth Military Medical University, Xi’an, Shaanxi China; 4 Department of Gastroenterology, Beijing Chao-Yang Hospital, Capital Medical University, Beijing, China; 5 Faculty of Life Science, Northwest University, Xi’an, Shaanxi China

**Keywords:** Cell invasion, Mechanisms of disease

Correction to: *Cell Death and Disease* 10.1038/s41419-018-1270-x, published online 15 January 2019

The original version of this article unfortunately contained a mistake. In Figure 2D of this article, the image of LV-GATA6 group using BGC823 cells at 0 h was accidentally duplicated the image belonging to its Control group when the authors prepared this figure in PowerPoint. This change does not affect any results or conclusions reported in this paper. The authors apologize for the error. The correct figure can be found below.