# Supplementary figures and images for: GATA6 suppresses migration and metastasis by regulating the miR-520b/CREB1 axis in gastric cancer
Source: Cell Death Dis. 2019 Jan 15;10(2):35. doi: 10.1038/s41419-018-1270-x (PMC6426848; doi:10.1038/s41419-018-1270-x)

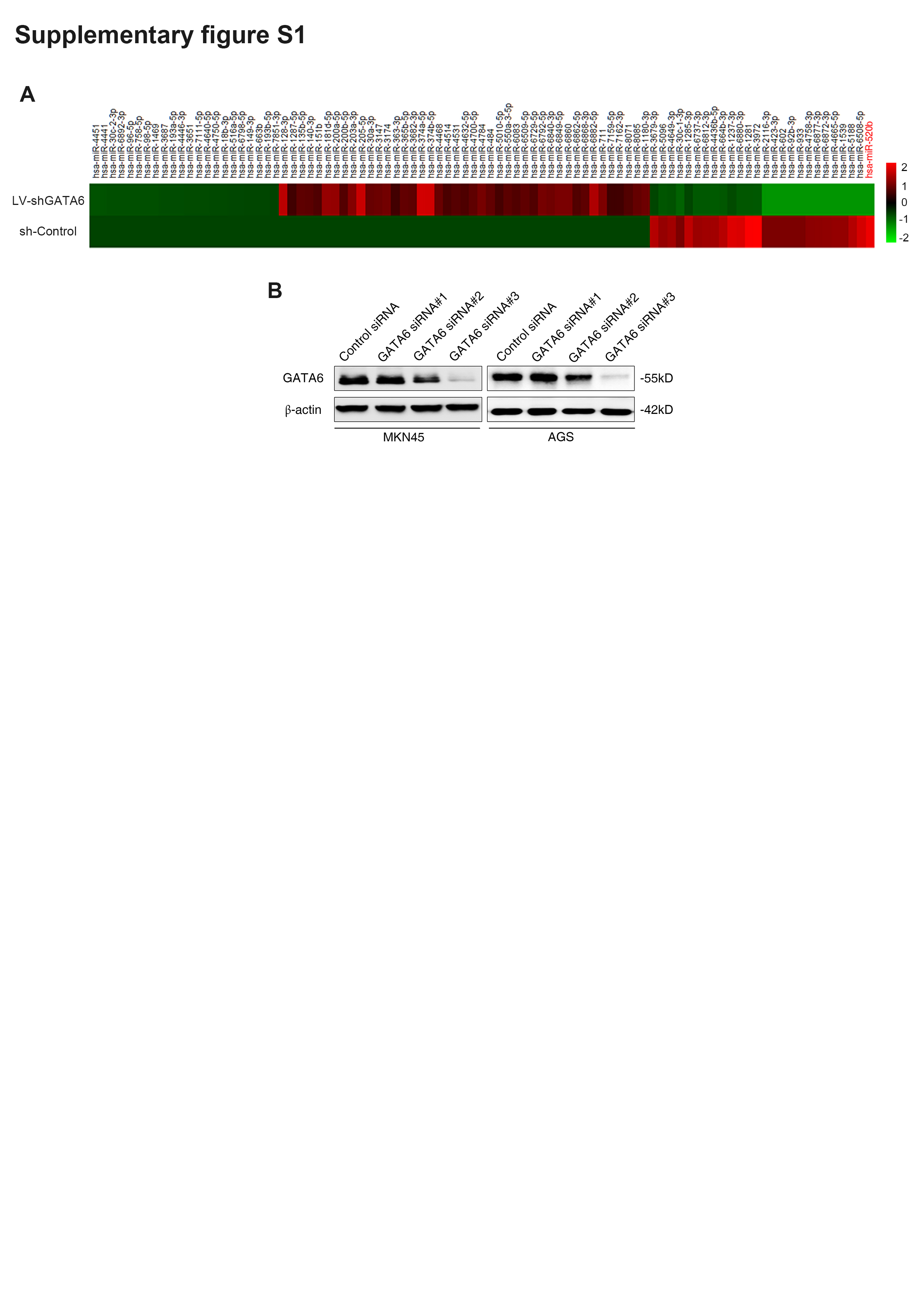

Supplement: Supplementary file 1 — supplementary figure S1 [file 41419_2018_1270_MOESM1_ESM.tif]
