# Supplementary material for: GATA6 suppresses migration and metastasis by regulating the miR-520b/CREB1 axis in gastric cancer
Source: Cell Death Dis. 2019 Jan 15;10(2):35. doi: 10.1038/s41419-018-1270-x (PMC6426848; doi:10.1038/s41419-018-1270-x)
Supplement: Supplementary file 2 — supplementary table 1 [file 41419_2018_1270_MOESM2_ESM.docx]

Supplementary Table S1 Primer sequences used in the study.

| Primer name | Primer sequence | Enzyme |
| --- | --- | --- |
| Primers for real-time PCR: |  |  |
| GATA6 sense: | 5’- TGCAATGCTTGTGGACTCTA -3’ |  |
| GATA6 antisense: | 5’- GTGGGGGAAGTATTTTTGCT -3’ |  |
| CREB1 sense: | 5’- GCTGCCTCTGGAGACGTACAA -3’ |  |
| CREB1 antisense: | 5’- GCTAGTGGGTGCTGTGCGA -3’ |  |
| MiR-520b sense | 5’- AAAGTGCTTCCTTTTAGAGGG -3’ |  |
| MiR-520b antisense | 5’- GCGAGCACAGAATTAATACGAC -3’ |  |
| ACTIN sense: | 5’- GGACTTCGAGCAAGAGATGG -3’ |  |
| ACTIN antisense: | 5’- AGCACTGTGTTGGCGTACAG -3’ |  |
| Primers for miR-520b promoter construct: | |  |
| (-2000/+500) miR-520b sense: | 5’- cgacgcgtTTGTTTGTGTGTGTGTGAGTGTATGTTTTATTTG -3’ | MluI |
| (-1097/+500) miR-520b sense: | 5’- cgacgcgtTGGTAATAAATTTGCAGAGAATG -3’ | MluI |
| (-265/+500) miR-520b sense: | 5’- cgacgcgtGGTATGTCTAGCAGGTATTGCTCTTTC -3’ | MluI |
| (-200/+500) miR-520b sense: | 5’- cgacgcgtACAAGGCTAACCTGCTGATTCTTTG -3’ | MluI |
| (+74/+500) miR-520b sense: | 5’- cgacgcgtTCAGAGTTGGATGATGGTGGGGTTCTG -3’ | MluI |
| Antisense: | 5’- ccgctcgagATAAGAGTATTCTCTGTGTTGGCCG -3’ | XhoI |
| Primers for miR-520b promoter site-directed mutagenesis: | |  |
| binding site 1 mutation sense: | 5’- AAGTTGATGCTcgcaTTGGTAATAAA -3’ |  |
| binding site 1 mutation antisense: | 5’- TTTATTACCAAtgcgAGCATCAACTT -3’ |  |
| binding site 2 mutation sense: | 5’- CCTTGGTTAAAtcgcAGGTATGTCTA -3’ |  |
| binding site 2 mutation antisense: | 5’- TAGACATACCTgcgaTTTAACCAAGG -3’ |  |
| binding site 3 mutation sense: | 5’- GATTGCCCCTTacgcAACAAGGCTAA -3’ |  |
| binding site 3 mutation antisense: | 5’- TTAGCCTTGTTgcgtAAGGGGCAATC -3’ |  |
| binding site 4 mutation sense: | 5’- CAGAGCATGCTcgctATCAGAGTTGG -3’ |  |
| binding site 4 mutation antisense: | 5’- CCAACTCTGATagcgAGCATGCTCTG -3’ |  |
| Primers used for ChIP with the miR-520b promoter: | |  |
| binding site 1 sense: | 5’- TCTAGAGGGAAGCACTTTCT -3’ |  |
| binding site 1 antisense: | 5’- ATTATAAGCATTCTCTGCAA -3’ |  |
| binding sites 2,3 sense: | 5’- ACAATGTATATTCTTCAGTA -3’ |  |
| binding sites 2,3 antisense: | 5’- TTCTGGAATCCAGAATATAA -3’ |  |
| binding site 4 sense: | 5’- TTAAGGAAGATTCCAACAAA -3’ |  |
| binding site 4 antisense: | 5’- AAGAGATCTAGGTTAAAAGT -3’ |  |
| Primers used for ChIP with the CREB1 promoter: | |  |
| Sense: | 5’- ACCTTCTCAGGTCCCCTTGC -3’ |  |
| Antisense: | 5’- CAGATGTCTCGTCTCAGAAC -3’ |  |
